# Anterior subcutaneous internal fixation for a complex vertical shear pelvic fracture with Morel-Lavallée lesion: a case report

**DOI:** 10.1093/jscr/rjag842

**Published:** 2026-09-20

**Authors:** Abdullah B Alshreef, Majd SaemAldahar, Dhafer S Almuffarh, Abdullah Alhazmi

**Affiliations:** College of Medicine, King Saud bin Abdulaziz University for Health Sciences, Makkah– Jeddah, King Abdulaziz Medical City, Jeddah 22384, Kingdom of Saudi Arabia; King Abdullah International Medical Research Centre, King Abdulaziz Medical City, Jeddah 22384, Kingdom of Saudi Arabia; Department of Surgery/Orthopedic section, King Abdulaziz Medical City, Jeddah 22384, Kingdom of Saudi Arabia; College of Medicine, King Saud bin Abdulaziz University for Health Sciences, Makkah– Jeddah, King Abdulaziz Medical City, Jeddah 22384, Kingdom of Saudi Arabia; King Abdullah International Medical Research Centre, King Abdulaziz Medical City, Jeddah 22384, Kingdom of Saudi Arabia; Department of Surgery/Orthopedic section, King Abdulaziz Medical City, Jeddah 22384, Kingdom of Saudi Arabia; College of Medicine, King Saud bin Abdulaziz University for Health Sciences, Makkah– Jeddah, King Abdulaziz Medical City, Jeddah 22384, Kingdom of Saudi Arabia; King Abdullah International Medical Research Centre, King Abdulaziz Medical City, Jeddah 22384, Kingdom of Saudi Arabia; Department of Surgery/Orthopedic section, King Abdulaziz Medical City, Jeddah 22384, Kingdom of Saudi Arabia

**Keywords:** Morel-Lavallée lesion, pelvic fracture, anterior subcutaneous internal fixation, INFIX, vertical shear injury, trauma

## Abstract

A 32-year-old male sustained a complex vertical shear pelvic fracture after a motor vehicle accident, associated with hemodynamic instability and a large Morel-Lavallée lesion involving the anterior pelvis and right thigh. Initial management included external fixation and percutaneous sacroiliac screw placement as part of damage-control orthopedics. Two weeks later, anterior subcutaneous internal fixation (INFIX) was performed to provide anterior pelvic stability while limiting further soft-tissue dissection. Postoperative imaging showed satisfactory reduction and implant positioning. The INFIX component was removed four months later after healing, with preserved pelvic stability. At 18 months, the patient was able to walk with a cane and had regained urinary continence, although bilateral foot drop persisted. This case highlights the role of soft-tissue condition in surgical decision-making for complex pelvic trauma.

## Introduction

Pelvic fractures are high-energy injuries associated with significant morbidity and frequent concomitant injuries, and their management is influenced by hemodynamic status, fracture pattern, and soft-tissue trauma [1, 2]. Several operative methods are available for anterior pelvic ring fixation, including external fixation, open reduction and internal fixation, and anterior subcutaneous internal fixation (INFIX), a minimally invasive technique used in selected unstable pelvic fractures [3, 4]. INFIX may be considered when anterior fixation is required and external fixation or open anterior approaches are less suitable [5]. The presence of a Morel-Lavallée lesion may further complicate surgical planning because of soft-tissue compromise and wound-related concerns [6]. This case report describes the use of INFIX in a complex vertical shear pelvic fracture complicated by a large Morel-Lavallée lesion.

## Case report

**Figure 1:**
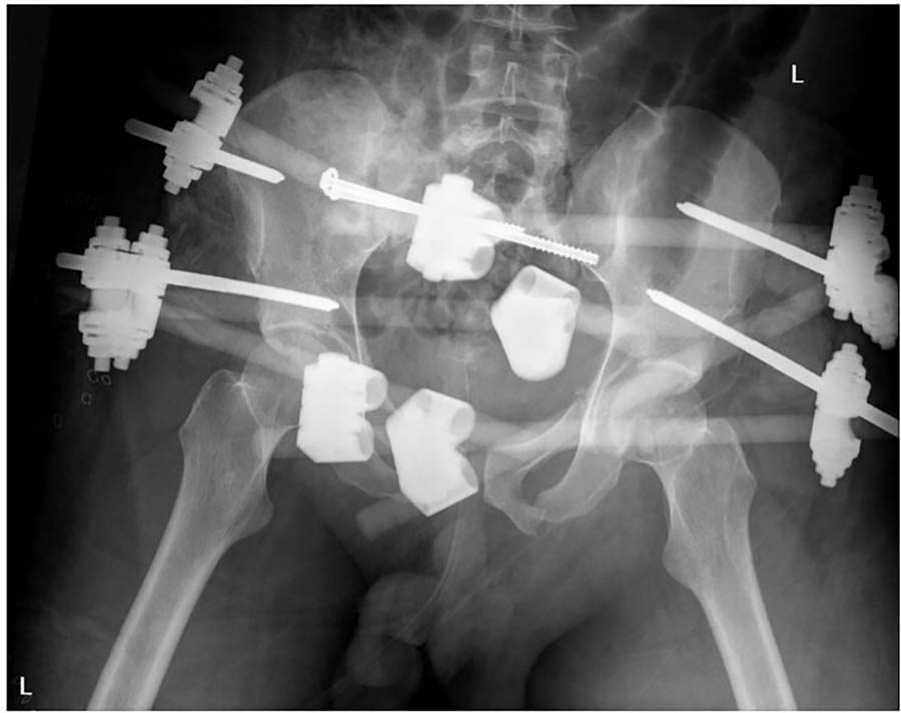
Pelvic radiograph showing stabilized pelvic fracture with an external fixator and supracetabular pins, in addition to sacroiliac screws.

**Figure 2:**
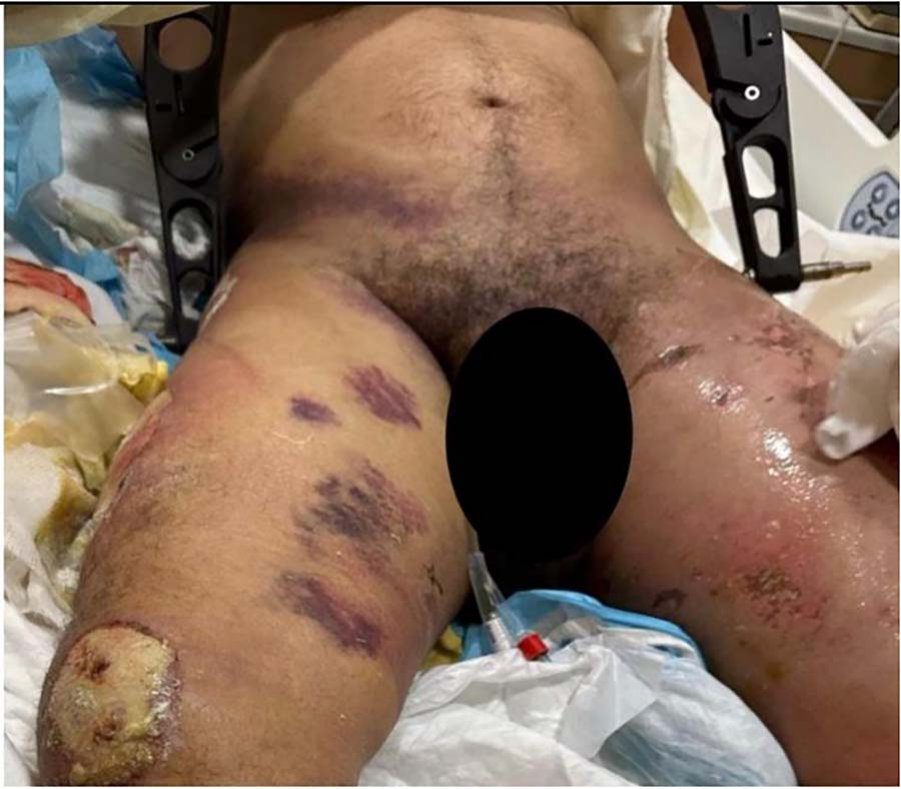
Clinical picture of the Morel-Lavallée lesion in the right thigh and part of the lower right abdomen.

A 32-year-old Asian male presented after a motor vehicle accident with a complex vertical shear pelvic fracture and initial hemodynamic instability. As part of damage-control orthopedic management, he underwent external fixation and percutaneous sacroiliac screw placement. Pelvic radiography showed stabilization with an external fixator, supracetabular pins, and sacroiliac screws (Fig. 1). He also had a large Morel-Lavallée lesion involving the anterior pelvis and right thigh (Fig. 2), raising concern for wound-related complications with open anterior fixation.

Neurological examination showed bilateral distal lower-limb deficits. On the left side, L4, L5, and S1 motor power was 0/5, with sensation 0/2 below the ankle. On the right side, ankle dorsiflexion was 1/5, while plantar flexion and big toe extension were 4/5, with intact sensation. Hip and knee range of motion could not be assessed because of pain. There were no signs of compartment syndrome.

Two weeks after injury, after stabilization and soft-tissue assessment, INFIX was performed. Under general anesthesia, the patient was positioned supine. Using C-arm fluoroscopic guidance, entry points were identified just above the bilateral anterior inferior iliac spines. Two 7.5 × 80 mm pedicle screws were inserted and connected with a subcutaneous carbon fiber rod while maintaining pelvic reduction. The posterior pelvic ring was also stabilized with iliosacral fixation. There were no intraoperative complications, and the estimated blood loss was ~100 mL.

**Figure 3:**
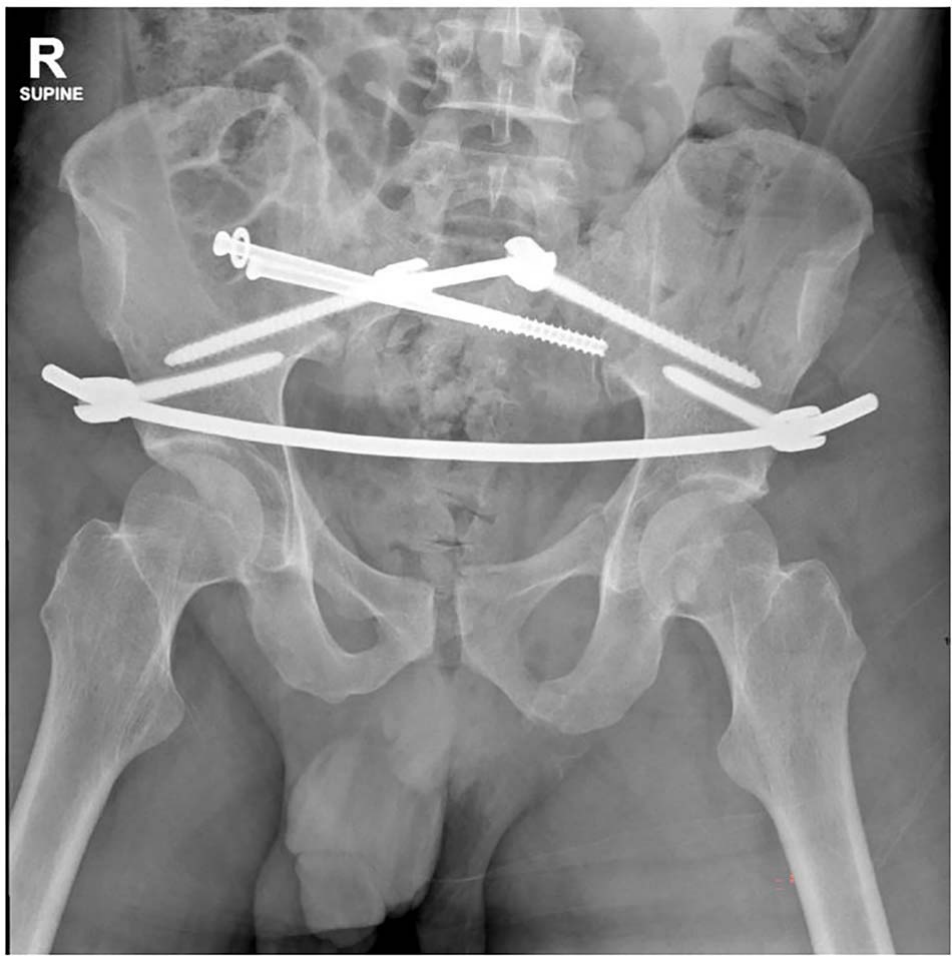
Post-surgical pelvic radiograph demonstrating INFIX technique for pelvic fracture, in addition to iliosacral fixation.

**Figure 4:**
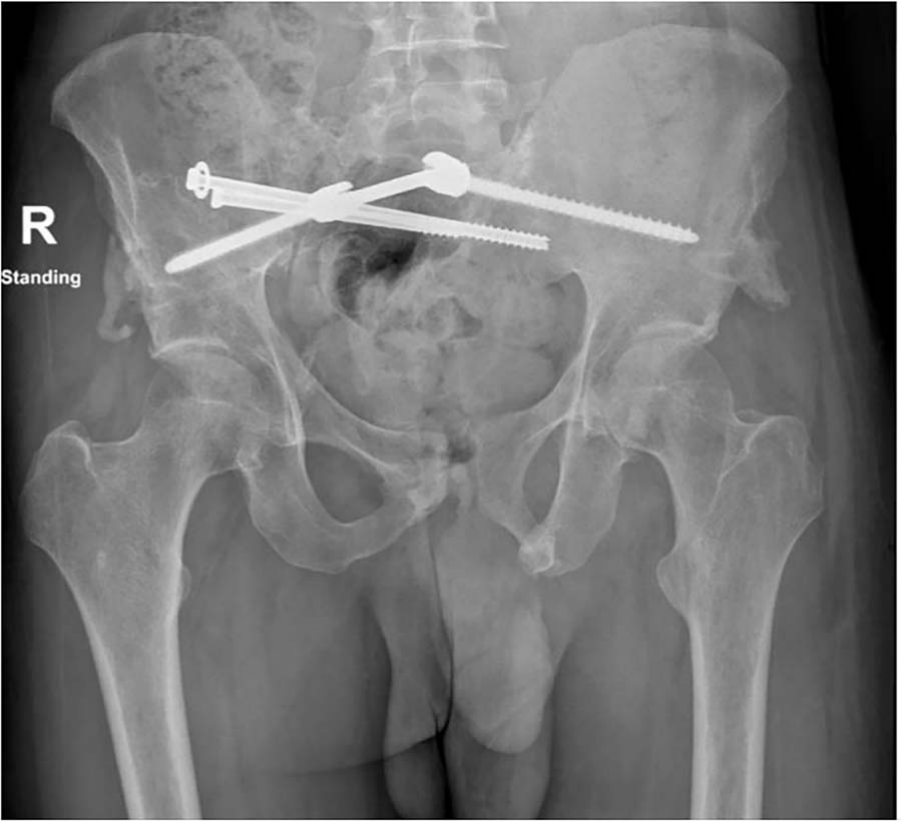
Pelvic radiograph after removal of the INFIX component, with remaining posterior pelvis implant.

Postoperative pelvic radiographs demonstrated satisfactory implant positioning and fracture reduction (Fig. 3). Four months postoperatively, the INFIX component was removed after healing, with preserved pelvic stability on follow-up imaging (Fig. 4). During follow-up, the patient improved but continued to have foot drop. At 18 months, left-sided sensation partially recovered to 1/2, with L5 and S1 motor power improving to 3/5 and L4 to 1/5. He was able to walk using a cane, regained urinary continence, and no longer required diapers. He was advised to continue rehabilitation and ankle-foot orthosis support.

## Discussion

Pelvic fractures are often associated with injuries to the peripelvic soft tissues, extremities, abdominal organs, and chest. In this case, the patient sustained a complex vertical shear pelvic fracture with a large Morel-Lavallée lesion. Morel-Lavallée lesions are closed traumatic degloving injuries caused by separation of the subcutaneous tissue from the underlying fascia, creating a potential space for blood, lymph, and necrotic fat collection [6]. These lesions are commonly associated with high-energy trauma and may influence surgical planning because of wound-related complications and surgical site infection, especially when located near the planned surgical approach [6].

Previous studies have described INFIX as a minimally invasive option for unstable anterior pelvic ring injuries when anterior fixation is required [3–5, 7]. The main value of this case is the use of INFIX in the setting of a large anterior pelvis and right thigh Morel-Lavallée lesion, where soft-tissue condition guided surgical decision-making. INFIX was selected to provide anterior pelvic stability while limiting additional anterior soft-tissue dissection.

The staged approach was also important. The patient was initially treated with external fixation and sacroiliac screw placement as damage-control management, followed by INFIX two weeks later after stabilization and soft-tissue assessment. This balanced the need for pelvic stability with the concern for wound-related complications. Implant-related complications should also be considered. Kumbhare *et al*. [5] reported implant failure, particularly when the implant is removed before adequate fracture healing. In this case, the INFIX component was removed four months postoperatively after healing, and pelvic stability was preserved. Vaidya *et al*. [8] reported that nursing care for INFIX patients may be less demanding than for patients treated with external fixation, particularly in ICU settings.

The clinical outcome should be interpreted cautiously. Although the patient regained urinary continence and was able to walk with a cane at 18 months, bilateral foot drop persisted. Reported INFIX-related complications include vascular injury, deep vein thrombosis, femoral nerve palsy, heterotopic ossification, lateral femoral cutaneous nerve injury, and operative site infection [5, 9–11]. Therefore, Morel-Lavallée lesions should not be interpreted as automatically favoring INFIX. Surgical planning should depend on fracture pattern, soft-tissue condition, patient stability, and surgeon experience.

This case suggests that INFIX may be considered as part of staged management in selected pelvic fractures with significant anterior soft-tissue compromise. In this patient, it provided anterior pelvic stability while limiting additional soft-tissue dissection.
